# Anemia and iron metabolism disorders after single anastomosis sleeve ileal (SASI) bypass. Is it a real problem?

**DOI:** 10.1007/s00423-024-03384-y

**Published:** 2024-06-21

**Authors:** Joanna Parkitna, Artur Binda, Agnieszka Gonciarska, Paweł Jaworski, Emilia Kudlicka, Krzysztof Barski, Karolina Wawiernia, Piotr Jankowski, Michał Wąsowski, Alina Kuryłowicz, Wiesław Tarnowski

**Affiliations:** 1 Department of General, Oncological and Bariatric Surgery, Centre of Postgraduate Medical Education, Orłowski Hospital, Czerniakowska 231, Warsaw, 00-416 Poland; 2 Department of General Medicine and Gerontocardiology, Centre of Postgraduate Medical Education, Orłowski Hospital, Czerniakowska 231, 00-416 Warsaw, Poland

**Keywords:** Bariatric surgery, Iron deficiency, SASI bypass, Obesity

## Abstract

**Purpose:**

SASI (single anastomosis sleeve ileal) bypass can lead to nutritional deficiencies, including disorders of iron metabolism and anemia. This study aims to evaluate the effect of SASI bypass on weight loss, anemia, and iron deficiency in patients with obesity during the follow-up period.

**Methods:**

This study is a retrospective analysis of prospectively collected data from patients who underwent SASI bypass at our hospital between January 2020 and February 2022.

**Results:**

The mean age of the patients was 42 years (range 22–58). The average duration of the follow-up period was 26 months. The mean percentage of excess weight loss (%EWL) was 90.1%, and total weight loss (%TWL) was 30.5%. During the postoperative observation period, anemia was identified in ten patients (25%), comprising 70% with normocytic anemia, 10% with microcytic anemia, and two macrocytic anemia cases (20%). Iron deficiency was observed in two patients (5%).

**Conclusion:**

SASI bypass is an effective bariatric procedure in weight loss outcomes. However, there may be an increased risk of anemia and iron metabolism disruptions associated with this procedure. The common limb length (250 vs. 300 cm) did not significantly impact hemoglobin, iron, TIBC, ferritin levels, or anemia incidence among patients undergoing SASI bypass. The decrease in postoperative ferritin levels signifies a depletion in tissue iron reserves, thereby emphasizing the necessity for surveillance of iron homeostasis parameters following SASI bypass.

## Introduction

Obesity is now considered a global epidemic. According to a World Health Organization (WHO) report, worldwide obesity has nearly tripled since 1975, with over 50% of the world’s population being overweight or with obesity [1]. Bariatric surgery is the most effective method for treating obesity [2]. Compared to non-surgical treatment options, bariatric surgery improves weight loss, obesity-associated medical problems, overall mortality, and quality of life [3].

According to a 2021 International Federation for the Surgery of Obesity and Metabolic Disorders (IFSO) report, the most commonly performed bariatric surgery is sleeve gastrectomy (SG), followed by Roux-en-Y gastric bypass (RYGB) [4]. Both procedures may be associated with nutritional deficiencies. The incidence of iron deficiency ranges from 18 to 53% after RYGB and from 1 to 54% after SG [3].

Single anastomosis sleeve ileal (SASI) bypass is a novel bariatric surgical technique combining mini gastric bypass elements and Santoro’s operation. This procedure involves a sleeve gastrectomy and a side-to-side gastro-ileal anastomosis [5]. Recent data indicates that the SASI bypass is an effective bariatric procedure for weight loss and addressing obesity-associated medical problems [6]. However, the restrictive and malabsorptive nature of SASI bypass can lead to nutritional deficiencies, including disorders of iron metabolism and anemia. This study aims to evaluate the effect of SASI bypass on weight loss, anemia, and iron deficiency in patients with obesity.

## Methods

This study is a retrospective analysis of prospectively collected data from patients with morbid obesity who underwent SASI bypass at our hospital between January 2020 and February 2022. Ethical approval for the study was obtained from the Regional Committee for Medical Research Ethics (313/2023). Clinical data were collected prospectively from the digital medical records, including laboratory test results and body weight parameters. Before the SASI bypass, each patient underwent a routine upper GI endoscopy. No pathological findings that could impact the occurrence of anemia and disturbances in iron metabolism post-surgery were identified in any case.

### Study population

Adult patients over 18 years of age of both sexes with morbid obesity and complete pre-operative data and at least 12 months of follow-up after SASI bypass were included in the study. Morbid obesity was defined as a BMI greater than 40 kg/m^2^ or greater than 35 kg/m^2^ with at least one associated co-morbidity. Patients eligible for SASI bypass specifically included those with one of the following criteria: type 2 diabetes, individuals experiencing upper gastrointestinal symptoms of GERD or displaying endoscopic evidence of erosive esophagitis graded A or B according to the Los Angeles classification, or those with a preference for malabsorptive bariatric procedures other than OAGB and RYGB. Candidates for SASI bypass also included individuals for whom a purely restrictive surgical approach might be ineffective, such as those with a strong preference for sweet foods or consuming at least 50% of the recommended daily carbohydrate intake in simple carbohydrates. All patients were informed about the procedure’s unique combination of benefits from sleeve gastrectomy and the malabsorptive aspect of the surgery. The investigational nature of the SASI bypass procedure was thoroughly explained to all candidates, emphasizing the uncertainties regarding midterm and long-term follow-up outcomes. Candidates were required to clearly understand these factors and provide written informed consent before surgery. Patients who were unwilling to accept the innovative nature of the procedure or unable to commit to the necessary post-operative follow-up were deemed ineligible for SASI bypass. Patients with commonly recognized contraindications to bariatric surgery, particularly malabsorptive procedures, were also not considered eligible for SASI bypass. Patients with erosive esophagitis at grade C or D according to the Los Angeles classification were considered eligible for RYGB.

Oral iron supplementation was administered for patients diagnosed with anemia or iron deficiency before surgery to achieve normal morphology parameters and iron levels. Surgical intervention was conducted after these parameters were normalized.

### SASI bypass surgical technique

**Fig. 1:**
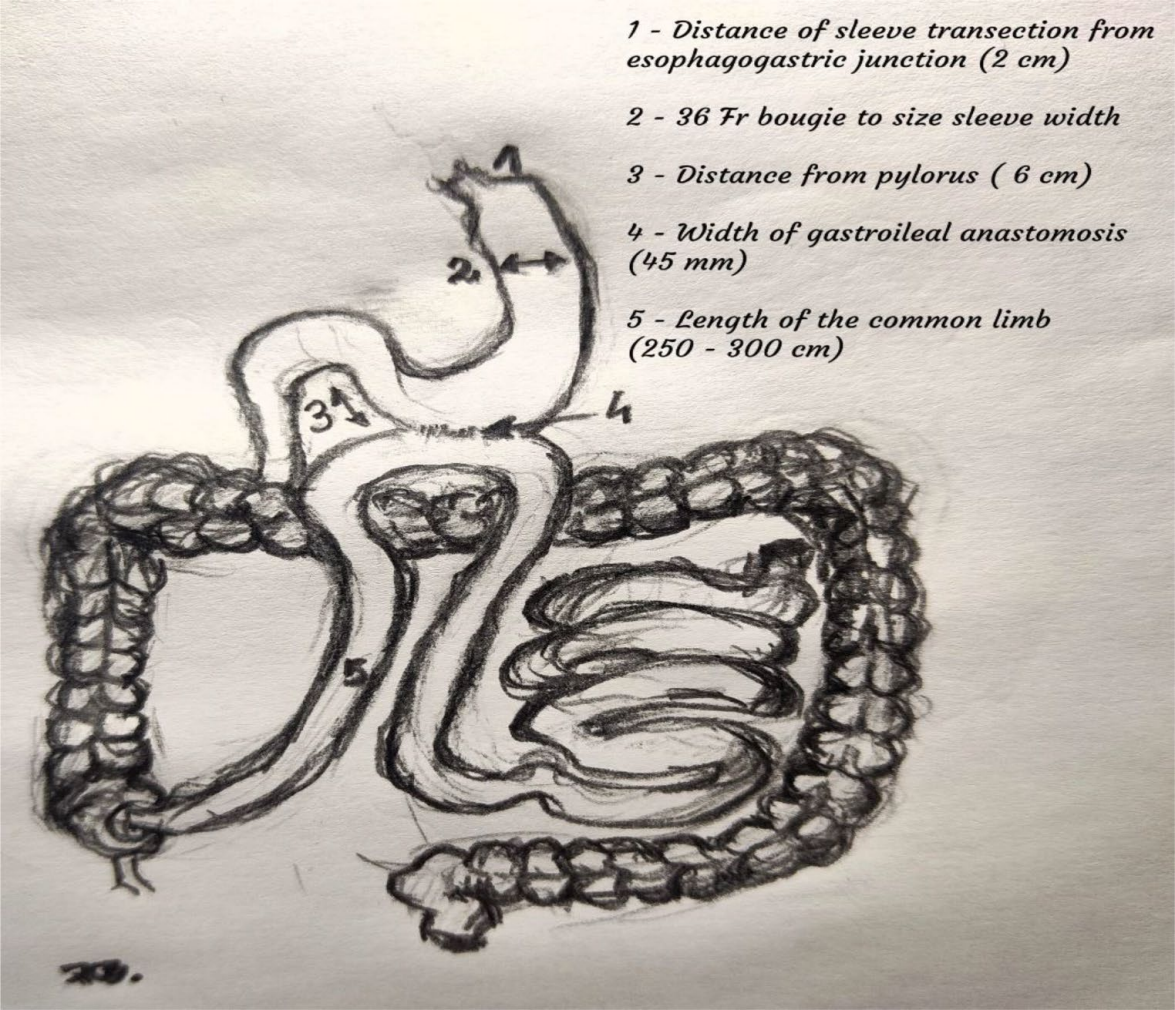
Single anastomosis sleeve ileal (SASI) bypass scheme

We utilized a standardized five-trocar technique under a pneumoperitoneum of 12 to 15 mmHg pressure. A 5 mm LigaSure Blunt Tip Laparoscopic Sealer/Divider (Medtronic) or Harmonic ACE (Ethicon Endo-Surgery) was employed to dissect the omentum from the greater curvature meticulously. Subsequently, a 36-Fr bougie was transorally inserted. Using the Signia™ powered stapler with Tri-Staple™ Reloads, 60 mm in purple and tan (Covidien, Medtronic, USA), we transected the stomach, initiating the incision 6 cm from the pylorus and extending it up to the angle of Hiss. This procedure phase was performed with the surgeon positioned between the patient’s legs, with assistants stationed on either side of the operating table. After completing the sleeve formation, the operating table was transitioned to a horizontal position, and the surgeon repositioned to the patient’s left side to begin the second phase of surgery. An additional 5 mm trocar was inserted in the lower left abdomen to aid in measuring the ileum. The Bauhin valve was identified, and the ileum was measured and brought to the gastric sleeve. The length of the common limb was set at either 250–300 cm, depending on the study period. An antecolic side-to-side gastro-ileal anastomosis was established using a 45-mm Tri-Staple™ Reload in tan (Medtronic). The stapler defect was closed using an absorbable V-Loc™ Wound Closure Device (Medtronic) in a single layer. Additionally, interrupted stitches were placed between the ileal loop and gastric sleeve to reinforce the anastomosis (Fig. 1).

As a routine, each patient was advised to use WLS Primo (FitForMe B. V.) for six months following surgery, starting the third week after the operation. For patients reporting intolerance to the supplement, primarily discomfort and nausea, modification of the intake method was recommended. In case of no improvement, the intake of readily available supplements with a complete set of minerals and vitamins with a similar composition was advised. The detailed composition of WLS Primo has been presented in Table 1.

**Table 1:** WLS Primo composition

Vitamins	Amount	RI^*^ [%]	Minerals	Amount		RI^*^ [%]
Vitamin A	1200 µg	150	Chromium		160 µg	400
Vitamin B1	3 mg	273	Iron	85 mg		607
Vitamin B2	3.5 mg	250	Iodine	225 µg		150
Niacine (B3)	32 mg	200	Copper	3 mg		300
Pantothenic acid (B5)	18 mg	300	Manganese	3 mg		150
Vitamin B6	1.12 mg	80	Molybdenum	112,4 µg		225
Biotin (B8)	100 µg	200	Selenium	105 µg		191
Folic acid (B9)	800 µg	400	Zinc	30 mg		300
Vitamin B12	500 µg	20,000				
Vitamin C	140 mg	175				
Vitamin D3	75 µg	1500				
Vitamin E	36 mg α-TE	300				

*RI = reference intake according to regulation (EU) 1169/2011

Data on weight loss, metabolic changes after surgery, and any potential issues were recorded before the procedure and during a follow-up period of at least 12 months. The diagnostic criteria for anemia were determined by identifying hemoglobin (Hb) levels below 12 g/dL for females and below 13 g/dL for males. Iron deficiency was diagnosed when iron levels fell below 37 µg/dL, regardless of the patient’s gender. Maximum weight refers to the highest weight a patient has ever reached, while maximum BMI indicates the highest body mass index recorded during a lifetime. Initial weight denotes the patient’s weight at the qualifying visit for bariatric surgery.

### Statistical analysis

Continuous quantitative variables were presented as mean ± standard deviation (SD) and minimum, maximum, median, first, and third quartiles. Descriptive statistics for quantitative variables were conducted. The Wilcoxon test was performed (due to the small study group size) to analyze the association between quantitative variables. For continuous variables, such as hemoglobin, iron, ferritin, and TIBC, a one-way analysis of variance (ANOVA) was used to evaluate differences between the two groups based on common limb length. Categorical variables were presented as numbers and group percentages, and compared using the chi-square test for independent samples. The required sample size was determined using a two-sample test power calculator with a test power of 90%, assuming *p* < 0.05, allocation ratio = 1, and alpha = 5. All statistical analyses were conducted using Statistica version 13.3 by StatSoft Polska. A p-value of < 0.05 was considered statistically significant.

## Results

We analyzed a cohort of 40 patients, four males and 36 females, who underwent SASI bypass surgery between January 2020 and February 2022. The mean age of the patients was 42 years (range 22–58). The average duration of the follow-up was 26 months. The preoperative patients’ characteristics are presented in Table 2.

**Table 2:** Preoperative characteristics of patients

Variable	Value
Age at operation (years), mean (range)	42 (22–58)
Female n (%)	36 (90%)
Male n (%)	4 (10%)
Type 2 diabetes mellitus n (%)	4 (10%)
Sweet preference in diet n (%)	12 (30%)
Gastroesophageal reflux disease n (%)	24 (60%)

### Weight loss outcomes

**Fig. 2:**
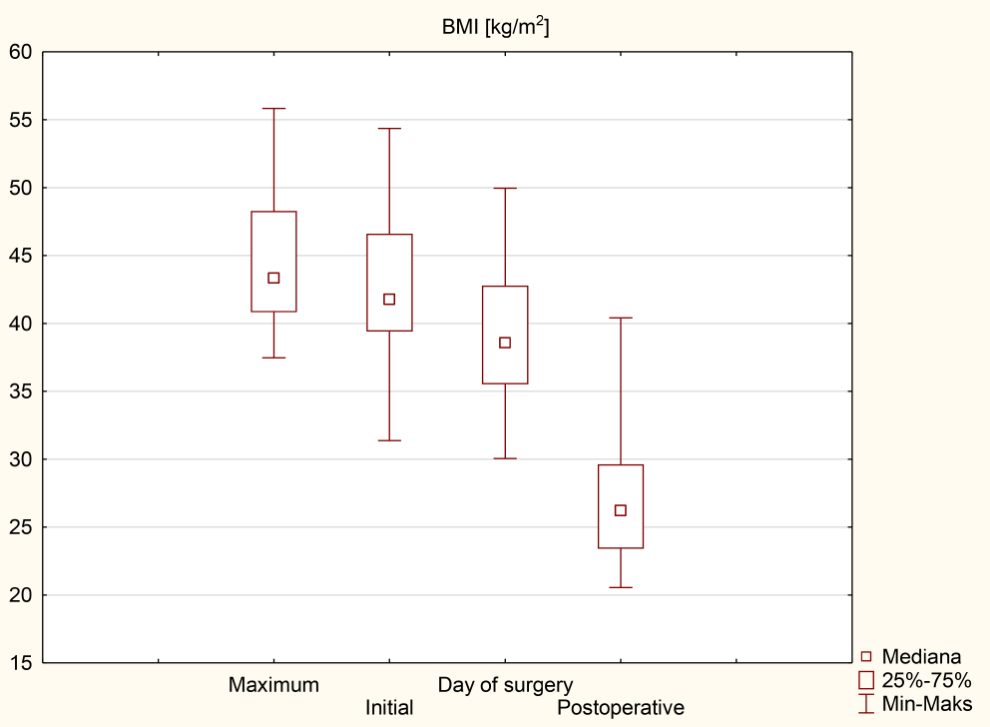
BMI loss

**Fig. 3:**
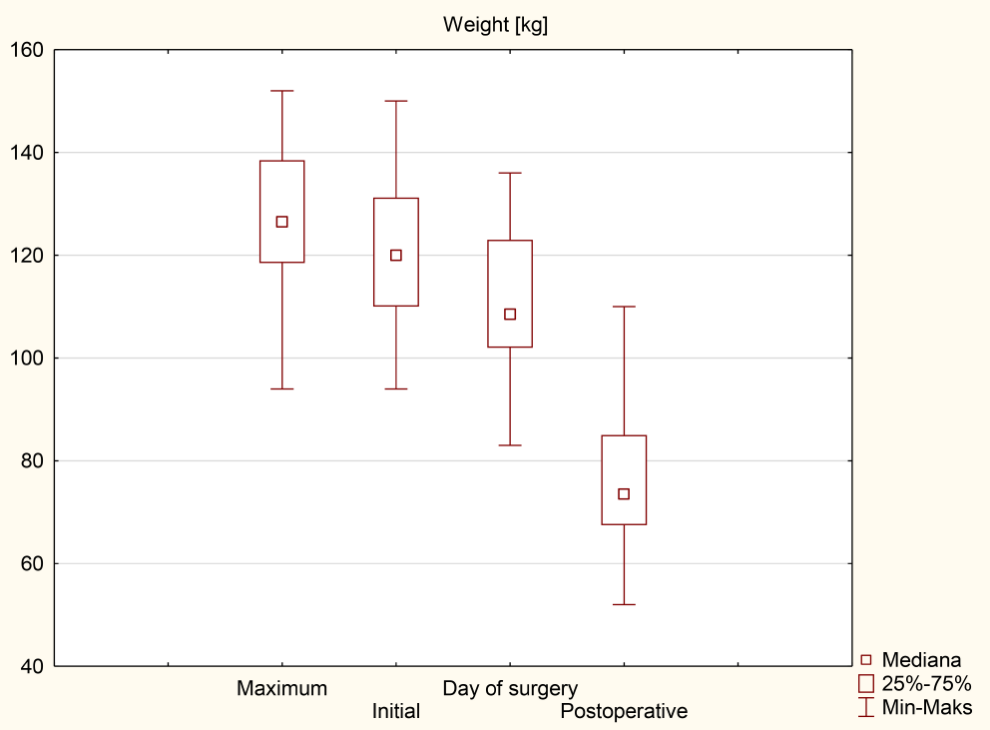
Weight loss

Patients’ initial average body weight was 120 (110-131.3) kg, and the initial mean BMI was 41.8 (39.4–46.6) kg/m^2^. On the day of surgery, weight and BMI were 108.5 (102–123) kg and 38.6 (35.5–42.8) kg/m^2^ respectively. The following weight loss parameters were recorded at the end of the follow-up: body weight was 73.5 (67.5–85) kg, BMI was 26.2 (23.4–29.6) kg/m^2^. The mean percentage of excess weight loss (%EWL) was 90.1%, and total weight loss (%TWL) was 30.5%. Detailed data are presented in Tables 3 and 4 as median (q1-q3) and Figs. 2 and 3.

**Table 3:** Weight loss parameters

Weight (kg)		Postoperative	*p*-value
Maximum^*^	126.5 (118.5-138.5)	73.5 (67.5–85)	< 0.001
Initial^**^	120 (110-131.3)		< 0.001
Day of surgery	108.5 (102–123)		< 0.001

Values are given as median (q1-q3), * the highest weight before surgery, **the surgery qualifying visit weight

**Table 4:** BMI loss parameters

BMI (kg/m^2^)		Postoperative	*p*-value
Maximum^*^	43.3 (40.8–48.3)	26.2 (23.4–29.6)	< 0.001
Initial^**^	41.8 (39.4–46.6)		< 0.001
Day of surgery	38.6 (35.5–42.8)		< 0.001

Values are given as median (q1-q3), * the highest BMI before surgery, **the surgery qualifying visit BMI

### Anemia and iron metabolism disorders

**Fig. 4:**
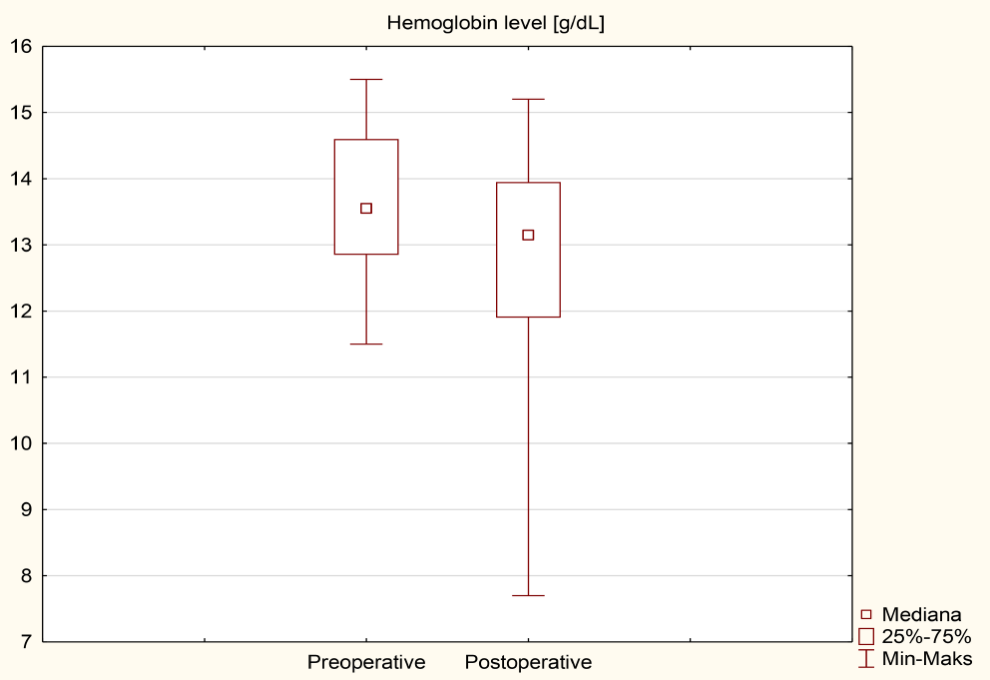
Comparison of hemoglobin level

**Fig. 5:**
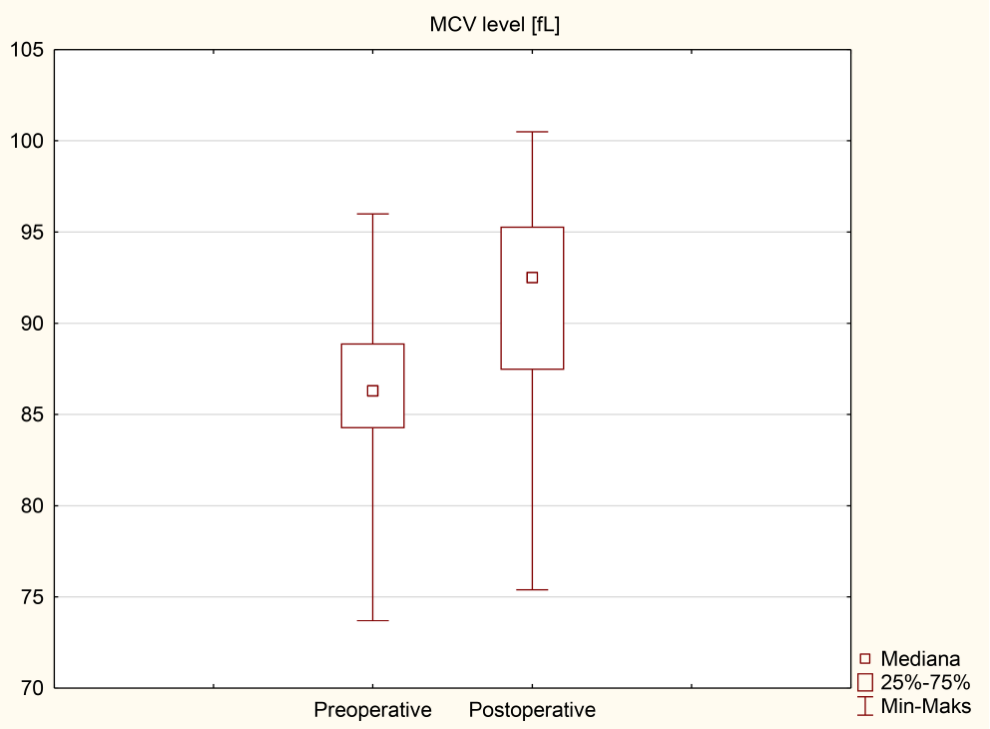
Comparison of MCV level

**Fig. 6:**
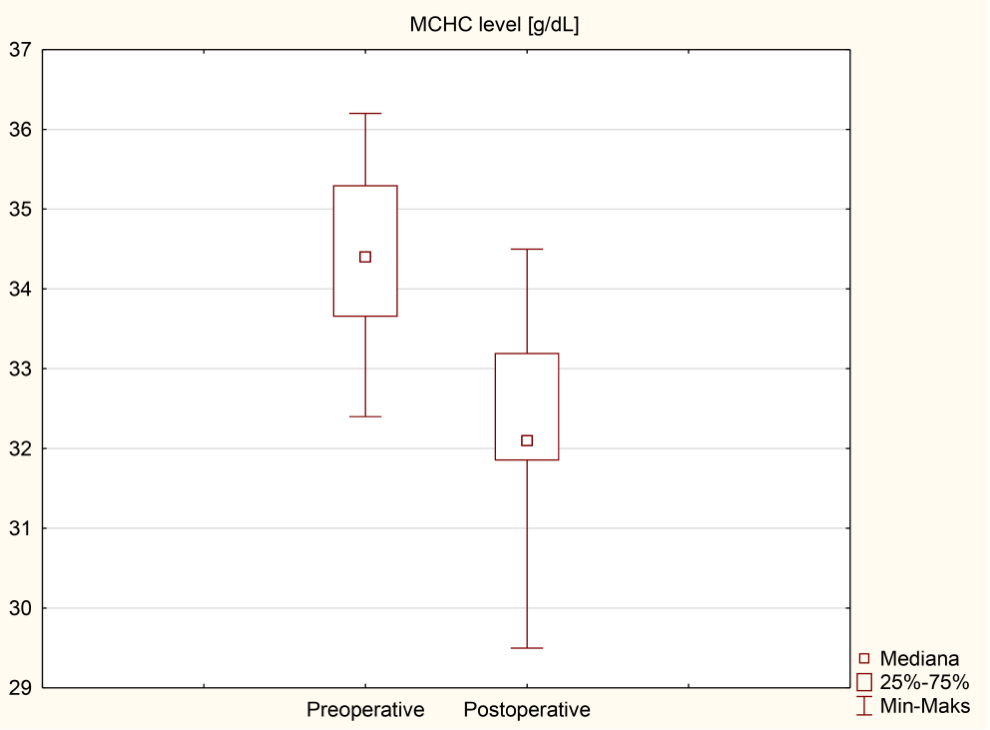
Comparison of MCHC level

**Fig. 7:**
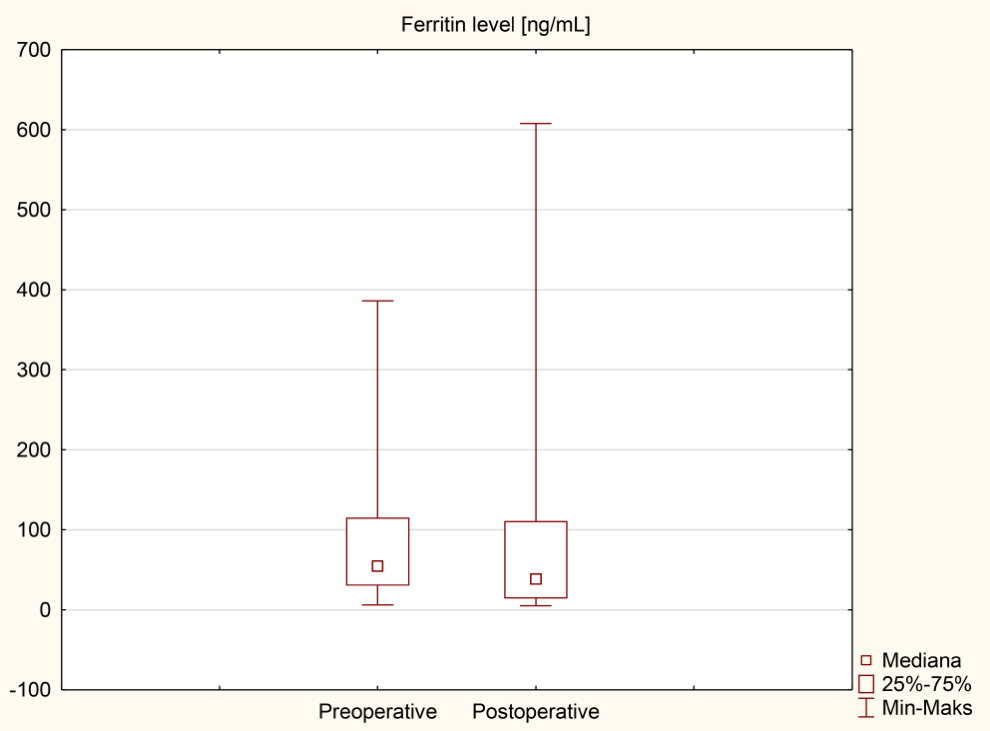
Comparison of ferritin level

The results of an analysis of individual iron metabolism parameters and blood count tests before and after surgery are presented in Table 5; Figs. 4, 5, 6 and 7 as median (q1-q3). A decrease in Hb levels from 13.6 (12.9–14.6) g/dL preoperatively to 13.2 (11.9–14) g/dL postoperatively (Fig. 4) and a decrease in MCHC levels from 34.4 (33.7–35.3) g/dL to 32.1 (31.9–33.2) g/dL (Fig. 6) were statistically significant (< 0.001). Statistical significance was observed in the case of an increase in MCV during the follow-up to 92.5 (87.5–95.3) fL compared to 86.3 (84.3–88.9) fL before the surgery in Fig. 5 (< 0.001). A decrease in ferritin levels was noted during follow-up; however, this change did not achieve statistical significance (*p* = 0.17) (Fig. 7). Before surgery, 5 out of 40 patients (12.5%) had overall anemia, which increased to 10 out of 40 patients (25%) during the follow-up (*p* = 0.15). Specifically, microcytic anemia was not observed before surgery but was present in 1 patient (2.5%) during the follow-up. Normocytic anemia was observed in 4 patients (10%) before surgery and in 7 patients (17.5%) during the follow-up (*p* = 0.33). Macrocytic anemia was present in 1 patient (2.5%) before surgery and in 2 patients (5%) during the follow-up (*p* = 0.56). Regarding iron deficiency, four patients (10%) before surgery and two patients (5%) during the follow-up period had iron deficiency (*p* = 0.39) (Table 6). De novo anemia occurred in 7 (17.5%) patients. In 3 (7.5%) patients, anemia persisted before and after surgery. Iron deficiency before surgery was present in 4 (10%) patients, and none of them showed iron deficiency after surgery. However, “de novo” iron deficiency was observed in 2 (5%) patients during the follow-up. At the end of the follow-up, the mean hemoglobin concentration for Group A (250 cm) was 12.6 ± 2.0 g/dL and 13.0 ± 1.4 g/dL for Group B (*p* = 0.42). Regarding the iron level at the end of the observation period, no statistically significant difference was observed, with values of 81.8 ± 34.3 µg/dL for Group A and 100.9 ± 62.4 µg/dL for Group B (*p* = 0.3). The TIBC concentration in Group A at the end of the observation period was 322.9 ± 54.5 µg/dL, and in Group B, it was 346 ± 44.4 µg/dL (*p* = 0.16). The ferritin concentration in Group A at the end of the follow-up was 89.6 ± 119 ng/mL; in Group B, it was 73.3 ± 117.6 ng/mL (*p* = 0.68). In the 250 cm group, the percentage of patients with anemia was 35.7%, while in the 300 cm group, it was 27%; the difference was not statistically significant (*p* = 0.85). The data has been presented in Table 7. Postoperative anemia detection prompted routine diagnostic assessments, including gastroscopy and colonoscopy. Among patients with postoperative anemia who underwent upper endoscopy, bile reflux was observed in 4, gastric mucosal inflammation in 1, and Grade A esophagitis according to the Los Angeles classification in 3 patients. Marginal ulceration was observed in one case. A colonoscopy revealed no abnormalities in any patient. Subsequently, each patient received oral iron supplementation with good tolerance. One patient required intravenous iron administration, and one patient received a blood substitute transfusion. Improvement was achieved in every patient.

**Table 5:** Iron metabolism parameters and blood count test results before and after the surgery

Variable	Preoperative	Postoperative	*p*-value
Hb [g/dL]	13.6 (12.9–14.6)	13.2 (11.9–14)	< 0.001
Hct [%]	39.9 (38–42)	40.8 (37.1–42.7)	0.893
MCH [pg]	30.1 (28.7–30.9)	30.4 (28.5–31.3)	0.36
MCHC [g/dL]	34.4 (33.7–35.3)	32.1 (31.9–33.2)	< 0.001
MCV [fL]	86.3 (84.3–88.9)	92.5 (87.5–95.3)	< 0.001
Fe [µg/dL]	77 (55–97)	87.5 (56.5–117)	0.18
TIBC [µg/dL]	341 (305.5-409.5)	338 (309-369.5)	0.47
Ferritin [ng/mL]	54.7 (30.2-115.3)	38.4 (14.1–111)	0.17

Values are given as median (q1-q3)

**Table 6:** The prevalence of anemia and iron deficiency

Variable	Patients with abnormal lab results		*p*-value
	Before surgery *n* (%)	During the follow up *n* (%)	
Overall anemia	5 (12.5)	10 (25)	0.15
microcytic	0 (0)	1 (2.5)	NA
normocytic	4 (10)	7 (17.5)	0.33
macrocytic	1 (2.5)	2 (5)	0.56
Iron deficiency	4 (10)	2 (5)	0.39

Anemia - Hb levels below 12 g/dL for females and below 13 g/dL for males, iron deficiency - iron levels below 37 µg/dL, NA - not applicable

**Table 7:** Impact of common limb length on anemia and iron deficiency

Variable	Group A 250 cm	Group B 300 cm	*p*-value
Hb [g/dL]	12.6 ± 2	13 ± 1.4	0.42
Fe [µg/dL]	81.8 ± 34.3	100.9 ± 62.4	0.3
TIBC [µg/dL]	322.9 ± 54.5	346 ± 44.4	0.16
Ferritin [ng/mL]	89.6 ± 119	73.3 ± 117.6	0.68

Values are given as mean ± SD

## Discussion

There is no ideal bariatric surgery, and the selection of an appropriate procedure should be tailored individually to each patient. Concerning weight loss parameters, the SASI bypass demonstrates excellent results [7]. The high efficacy of the SASI bypass in weight loss was also corroborated in the present study, with a %EWL of 90.1% and a %TWL of 30.5% during follow-up.

Enani et al. demonstrated an incidence of anemia after RYGB at 14.8%, compared to 1.6% after SG [8]. Another previous investigation, with varying follow-up periods, reported a high frequency of anemia ranging from 18 to 35% after RYGB [9]. As for iron deficiency, in the Enani study, the incidence after RYGB was 22.5%, and after SG, it was 12.4% [8]. The wide range of reported incidences of iron deficiency and anemia after LSG and RYGB is a complex issue influenced by several factors. These include variations in definitions of iron deficiency and anemia, patient demographics, differences in surgical techniques, variations in follow-up duration, and discrepancies in study design and methodology [3]. In this study, we aim to unravel this complexity and provide a clearer understanding of the extent of the problem regarding SASI bypass. Mahdy et al. conducted a study comparing RYGB to SASI bypass, where they also demonstrated that RYGB is associated with a higher risk of anemia and iron deficiency compared to SASI bypass. This study achieved a postoperative Hb level of 12.8 ± 1.66 g/dL in a group of 46 patients, compared to 12.9 ± 1.9 g/dL before the surgery, which is similar to our findings [10]. Another work covering a 3-year follow-up in 61 patients shows a statistically significant decrease in Hb and ferritin levels [11].

Anemia in patients with obesity poses a significant challenge, as observed in some patients before surgery. In our study, anemia was diagnosed in five patients (12.5%) before surgery. Conversely, anemia following bariatric surgery can be attributed to nutritional deficiencies stemming from reduced food intake, dietary habits, impaired iron absorption, diminished gastric acid production, decreased gastric intrinsic factor secretion, and rapid weight loss [3]. According to the Polish Central Statistical Office, the reproductive age in Poland is considered to be between the ages of 15 and 49. In our study, 26 women were of reproductive age, and no patient reported heavy menstrual periods, which could indicate a higher incidence of anemia in this group of patients. The findings from the present study indicate that the prevalence of overall anemia increased from before surgery to during the follow-up period. However, this difference was not statistically significant (*p* = 0.15). Specifically, there was a slight increase in the prevalence of normocytic anemia from before surgery to during the follow-up, but again, this difference was not statistically significant (*p* = 0.33). Microcytic and macrocytic anemia showed minimal changes. Regarding iron deficiency, the data revealed a decrease in prevalence from before surgery to during the follow-up period, although this difference was not statistically significant (*p* = 0.39). These results suggest that while SASI bypass may have some impact on the prevalence of anemia and iron deficiency, the changes observed were not statistically significant. Risk of anemia, can be attributed to the surgery’s restrictive and malabsorptive nature. It appears that iron deficiency following the SASI bypass should be less prevalent than in RYGB, primarily because the SASI bypass preserves the passage through the main sites of iron absorption, namely the duodenum and jejunum [12]. Our findings show statistically significant changes in several iron metabolism parameters following surgery. Hemoglobin levels decreased significantly from a median of 13.6 g/dL (preoperative) to 13.2 g/dL (postoperative) (*p* < 0.001), indicating a potential impact on red blood cell production. Mean corpuscular volume (MCV) significantly increased from 86.3 fL to 92.5 fL (*p* < 0.001), suggesting changes in erythrocyte size. Additionally, mean corpuscular hemoglobin concentration (MCHC) significantly decreased from (*p* < 0.001), indicating alterations in hemoglobin content per red blood cell. These findings suggest that SASI bypass surgery may influence iron metabolism, potentially affecting erythropoiesis and red blood cell characteristics. The observed increase in iron levels and total iron-binding capacity (TIBC) levels after surgery can be attributed to the standard micro and macronutrient supplementation recommended following bariatric surgery. Particular attention should be given to the marked decrease in ferritin levels, which may indicate iron depletion and necessitate supplementation both orally and intravenously within hospital settings, incurring higher costs. The guidelines provided by the American Society of Metabolic and Bariatric Surgery (ASMBS) recommend diagnosing iron deficiency before bariatric surgery and monitoring it every three months during the first year following the surgery, with subsequent annual check-ups. This monitoring is typically conducted by measuring serum iron, serum transferrin, and TIBC [13]. Guidelines for iron supplementation after RYGB and SG are well-established [8]. However, it is worth noting that no specific recommendations are available for iron supplementation following the SASI bypass procedure.

The length of the common limb is one of the critical factors influencing the outcomes of SASI bypass. The common limb length could impact nutritional status, rate of complications, and weight loss outcomes [14]. The extent of the common loop formed during the SASI bypass can range between 250 and 350 cm [7, 14, 15]. A shorter common limb may increase malabsorption and enhanced weight loss but could also elevate the risk of complications [14, 16]. The decision-making process for the common limb length during SASI bypass should consider the balance between achieving adequate weight loss and minimizing the risk of nutritional complications such as protein-caloric malnutrition or iron metabolism disorders. Varied by the surgical period in the present study, the common limb length was 250 cm for the first 14 patients, which increased to 300 cm for the subsequent 26 patients due to our observations regarding the relatively high percentage of patients with nutritional deficiencies, when creating a common limb of 250 in length, as well as the gradually emerging research findings confirming our observations in the literature. However, in the present study, we did not find a significant influence of common limb length, whether 250 vs. 300 cm, on hemoglobin levels (*p* = 0.42), iron (*p* = 0.3), ferritin (*p* = 0.68), TIBC concentration (*p* = 0.16), or the percentage of patients with anemia (*p* = 0.85) at a mean follow-up period of 26 months.

Despite the potential adverse effects on iron metabolism parameters in some patients, it is worth emphasizing that SASI bypass has promising outcomes, particularly in addressing metabolic disorders such as type 2 diabetes [5, 14–18]. As a malabsorptive surgery, SASI bypass may benefit individuals with a preference for sweets. According to available reports, SASI bypass has a beneficial effect on resolving symptoms associated with GERD [14, 15, 17]. Considering this, SASI bypass is among our alternatives for patients in whom a purely restrictive surgical approach, such as LSG, might be ineffective and with a preference for malabsorptive bariatric procedures other than OAGB and RYGB.

This study has limitations including its retrospective nature, hindering causal assessment and definitive conclusions. Moreover, the predominantly female sample limits generalizability to males. We acknowledge that a sample size of 40 cases may raise concerns about statistical power and the ability to draw robust conclusions. The absence of a control group limits comparative analysis, though our focus was on the impact of SASI bypass on iron metabolism. Conducted at a single center with a small cohort, outcomes may be influenced by variable common limb lengths (250–300 cm). Our center now employs a 350 cm common limb to mitigate malnutrition risk. Larger, prospective studies with longer follow-up are needed for precise conclusions.

## Conclusions

The SASI bypass emerges as an effective bariatric procedure with favorable weight loss outcomes. However, there may be an increased risk of anemia and iron metabolism disruptions associated with this procedure. The common limb length (250 vs. 300 cm) did not significantly impact hemoglobin, iron, TIBC, ferritin levels, or anemia incidence among patients undergoing SASI bypass. The decrease in postoperative ferritin levels signifies a depletion in tissue iron reserves, thereby emphasizing the necessity for surveillance of iron homeostasis parameters following SASI bypass.

## Data Availability

Data is provided within the manuscript or supplementary information files.
